# User‐Interactive Thermotherapeutic Electronic Skin Based on Stretchable Thermochromic Strain Sensor

**DOI:** 10.1002/advs.202001184

**Published:** 2020-06-08

**Authors:** Giwon Lee, Geun Yeol Bae, Jong Hyun Son, Siyoung Lee, Seong Won Kim, Daegun Kim, Seung Goo Lee, Kilwon Cho

**Affiliations:** ^1^ Department of Chemical Engineering Pohang University of Science and Technology Pohang 37673 Korea; ^2^ Department of Chemistry University of Ulsan Ulsan 44610 Korea

**Keywords:** electronic skins, silver nanowires, strain sensors, thermochromic composites, wrinkles

## Abstract

User‐interactive electronic skin (e‐skin) with a distinguishable output has enormous potential for human–machine interfaces and healthcare applications. Despite advances in user‐interactive e‐skins, advances in visual user‐interactive therapeutic e‐skins remain rare. Here, a user‐interactive thermotherapeutic device is reported that is fabricated by combining thermochromic composites and stretchable strain sensors consisting of strain‐responsive silver nanowire networks on surface energy‐patterned microwrinkles. Both the color and heat of the device are easily controlled through electrical resistance variation induced by applied mechanical strain. The resulting monolithic device exhibits substantial changes in optical reflectance and temperature with durability, rapid response, high stretchability, and linear sensitivity. The approach enables a low‐expertise route to fabricating dynamic interactive thermotherapeutic e‐skins that can be used to effectively rehabilitate injured connective tissues as well as to prevent skin burns by simultaneously accommodating stretching, providing heat, and exhibiting a color change.

## Graphical abstract

User‐interactive thermotherapeutic electronic skin (e‐skin) is fabricated by combining thermochromic composites and stretchable strain sensors consisting of strain‐responsive silver nanowire networks on surface energy‐patterned microwrinkles. Both the color and heat of the device are easily controlled through electrical resistance variation induced by external mechanical strain. This e‐skin can be applied to effectively rehabilitate a connective tissue injury, being avoided from skin burns.

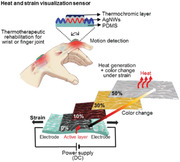

User‐interactive devices change their color, transparency, and shape in response to external stimuli (i.e., strain,^[^ ^1^, ^2^, ^3^ ^]^ pressure,^[^ ^4^, ^5^, ^6^ ^]^ chemical,^[^ ^7^, ^8^ ^]^ light,^[^ ^9^ ^]^ and temperature^[^ ^10^ ^]^), which enables users to be visually aware of the stimuli. These visual changes provide a versatile platform for devices such as electronic skin (e‐skin),^[^ ^11^ ^]^ smart windows,^[^ ^12^, ^13^ ^]^ and soft robotics^[^ ^14^ ^]^ to interact with the user under widely varying stimuli. Most importantly, user‐interactive e‐skins can be attached to movable parts and can react to environmental stimuli. Thus, e‐skins have numerous potential applications, including human motion detection,^[^ ^15^ ^]^ health monitoring,^[^ ^8^ ^]^ and human–machine interfaces.^[^ ^6^ ^]^ Substantial improvements have also been achieved in skin‐attachable therapeutic devices that can dynamically perform real‐time therapy of a human body.^[^ ^16^, ^17^, ^18^ ^]^ However, visual user‐interactive therapeutic devices have not yet been demonstrated, and previously developed therapeutic devices have been mostly fabricated through sophisticated integration of individual sensors and therapy components. A simple and controllable method that enables the fabrication of user‐interactive therapeutic devices is, thus, a desirable goal.

Thermotherapy is one of the simplest methods of alleviating and treating connective tissue injuries. To effectively treat and rehabilitate injured connective tissue, heat and stretch must be applied simultaneously.^[^ ^19^ ^]^ Therefore, researchers have recently developed stretchable thermotherapeutic devices based on stretchable conducting materials.^[^ ^16^, ^20^, ^21^ ^]^ For example, Ouyang's group fabricated a highly stretchable electrothermal heater using composites of intrinsically conductive poly(3,4‐ethylenedioxythiophene):poly(styrene sulfonic acid), elastomeric waterborne polyurethane, and reduced graphene oxide; their heater can generate approximately the same amount of heat under tensile strains as large as 30%.^[^ ^17^ ^]^ Kim's group developed a soft, thin, and stretchable heater using stretchable nanocomposites of silver nanowires (AgNWs) and thermoplastic elastomers, which enabled effective heat transfer to curvilinear joints even during motion.^[^ ^18^ ^]^ Despite these achievements, the aforementioned devices only demonstrated heat control via an applied electrical source or an additional circuit of microcontroller units. They do not exhibit visual changes in response to heat, which is critical characteristic of user‐interactive thermotherapeutic devices. Human skin cannot recognize absolute temperature and adapts easily to persistent heat.^[^ ^22^ ^]^ The long‐term application of heat to human skin causes low‐temperature burns: 44 °C (6 h), 45 °C (3 h), 48 °C (15 min), and 52 °C (1 min).^[^ ^23^ ^]^ Consequently, the development of an easily controlled thermotherapeutic visualization device in a stretchable form is highly desirable.

Here, we present a user‐interactive temperature visualization heater comprising a thermochromic film on a stretchable strain sensor. To the best of our knowledge, although stretchable devices based on AgNW networks and structured elastomer substrates have been extensively studied as stretchable strain sensors^[^ ^24^ ^]^ or heaters,^[^ ^18^ ^]^ no attempts to integrate a highly stretchable strain sensor with a heater for thermotherapy have been reported. The stretchable heater developed in the present work consists of highly percolated AgNWs on a wrinkled poly(dimethylsiloxane) (PDMS) film. When tensile strain is applied, the geometry of the wrinkles and the percolation of the AgNWs change, increasing the film's resistance. This behavior generates a large amount of heat and transfers it to the thermochromic layer to induce color changes. The device can withstand up to 100% strain and endure 1000 repeated mechanical strain (50%) cycles under stretch/release conditions. These smart devices were attached to human skin, where they function as thermotherapy visualization devices to effectively control various amounts of heat transfer depending on the degree of human motion while simultaneously preventing skin burns. This versatile device can treat areas ranging from small finger joints to large wrist joints.

**Figure** **1**a shows a schematic of the integrated stretchable device, which induces heat generation and color change under tensile strain for thermotherapeutic rehabilitation. When the stretchable device is mounted on a human hand and then deformed by movement of the finger or wrist joints, its electrical resistance changes, inducing changes in its heat generation and color. As the tensile strain in the joint increases, the device generates more heat, and users can detect color changes from dark to bright. In our user‐interactive smart device, a stimuli visualization layer is stacked vertically on the heat generation layer to simultaneously activate heat generation and visualization. As shown in the circuit diagram (Figure 1b), a constant current (*I*) is supplied to the stretchable device through a power supply to enable joule heating. The variable (*R*_active_) and fixed (*R*_electrode_) resistances of the single device were simply induced on the wrinkled film by tuning the AgNW deposition conditions.

**Figure 1:**
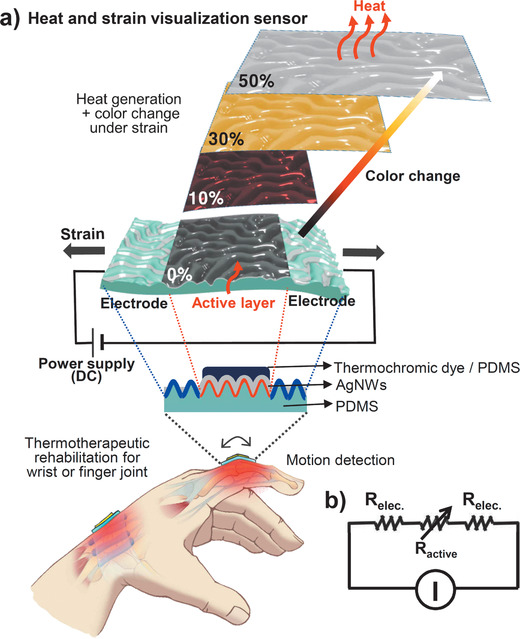
a) Schematic diagram of the integrated stretchable device inducing heat generation and color change under tensile strain for thermotherapeutic rehabilitation, and working mechanism. b) Circuit diagram for the stretchable device.

Our experimental procedure is illustrated in **Figure** **2**a. This procedure can be divided into three parts: chemical and physical modification of the PDMS substrate, deposition and alignment of the AgNWs, and coating of the thermochromic layer. The random wrinkled substrate with an average wavelength of 50 µm and an amplitude of 15 µm was fabricated by mechanically stretching a PDMS film, followed by UV‐ozone (UVO) exposure and strain release (Figure S1, Supporting Information). The wrinkled surface was selectively hydrophobized using trichloro(1*H*,1*H*,2*H*,2*H*‐perfluorooctyl)silane and a mask of polyethylene terephthalate (PET) film (see Supporting Information for more details). The hydrophobic surfaces were selectively formed around the hydrophilic surfaces located in the center of the wrinkled substrate. To characterize the surface chemical atomic states of the wrinkled PDMS substrate influenced by the UVO and silane treatments, we used X‐ray photoelectron spectroscopy (XPS) to analyze the variations in the C 1s spectra (Figure 2b). A C‐F peak is observed in the spectrum of the hydrophobic surface after the –CF_3_ treatment, which lowers the surface energy of the PDMS wrinkles. An AgNW solution (0.01 g mL^–1^ in water) was then dropped onto the wrinkled substrate, including the hydrophobic and hydrophilic surfaces. As evaporation proceeded, the AgNWs (40 nm in diameter, 20–60 µm in length) aligned along the troughs of the hydrophobic wrinkled patterns and randomly deposited throughout the hydrophilic wrinkled substrate. As a result, the active layer with a change in resistance under tensile strain was arranged in parallel between two electrodes. Finally, a thermochromic film comprising a dye (leuco dye, microcapsules 1–10 µm in diameter, Nano I&C) and PDMS was spray‐coated onto the randomly deposited AgNW film.

**Figure 2:**
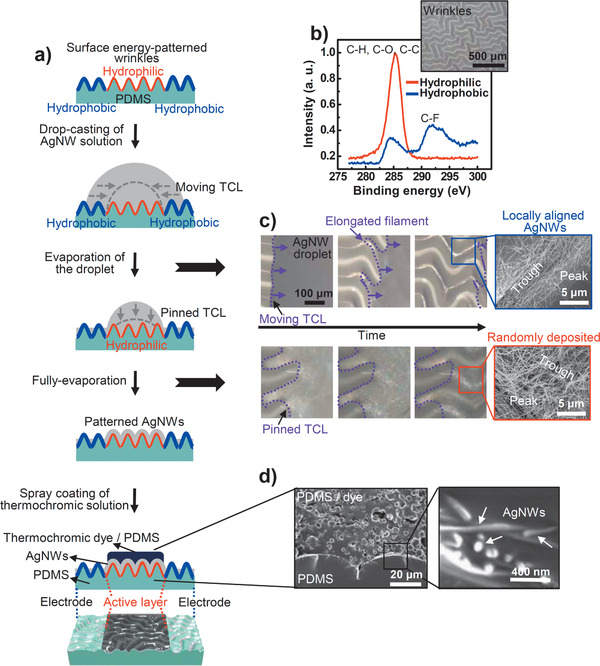
a) Scheme describing the fabrication process comprising chemical and physical modification of the PDMS substrate, deposition and alignment of AgNWs, and coating of the thermochromic layer. b) XPS analysis of partially patterned surface of wrinkled PDMS substrate. Inset: optical microscopy (OM) image of the PDMS wrinkles. c) Sequential OM images of an AgNW‐containing evaporating droplet on the hydrophobic and hydrophilic wrinkled surface. Insets are SEM images of the evaporation‐induced AgNW morphologies (locally aligned and randomly deposited AgNWs) on a PDMS wrinkled substrate with patterned surface energy. d) Cross‐sectional SEM images of the integrated device.

To gain insight into the process governing the spontaneous patterning of AgNWs during the drying of droplets on the wrinkled substrate, we investigated the sequential three‐phase contact line (TCL) dynamics of the drying droplets using optical microscopy (Figure 2c; Figures S2 and S3, Supporting Information). As the droplet of AgNW solution evaporated, the TCL on the hydrophobic surface moved toward the droplet center because of the weak interaction between the liquid and the solid substrate. After the TCL of the droplet moved, the elongated filament morphology of the solution along the wrinkled substrate remained intact, governed by the balance between friction (i.e., pinning) and capillary (i.e., depinning) forces.^[^ ^25^ ^]^ However, when the drying droplet reached the hydrophilic surface, strong interaction with the substrate fixed the TCL until the solution was completely evaporated. The sequential TCL dynamics of the droplet and high specific gravity of the AgNWs selectively aligned (only located on the trough of the wrinkle) or evenly deposited (randomly covered on every part of the wrinkle) AgNWs over a large area depending on the surface energy. The as‐prepared device was fabricated using strain‐responsive AgNW networks and thermochromic dyes in an intrinsically stretchable PDMS elastomer, which acts as a substrate and binder. Remarkably, each layer of the all‐PDMS‐based devices produced no interfacial separation between the AgNWs and thermochromic dyes (Figure 2d); therefore, these monolithic PDMS composites are especially useful for ultrastable strain‐responsive devices because of their structural robustness with respect to interfacial failure under external strain.

The electrical properties of the AgNW arrays were affected by their deposition geometries on the wrinkled substrate. **Figure** **3**a shows the normalized resistances (Δ*R*/*R*_0_, where *R*_0_ is the initial resistance) of each AgNW array as the tensile strain is increased from 0% to 100%. The change in resistance for the randomly deposited AgNWs increases proportionally with increasing the tensile strain. For the selectively aligned AgNWs, however, the resistance is relatively insensitive to stretching^[^ ^26^ ^]^ because of the wavy structure of the locally higher‐density AgNW bundles; furthermore, the long structural contours can absorb stress by extending the wavy structure without accumulating mechanical stress. These results indicate that our solution‐based fabrication method can be used to adjacently create both strain‐sensitive (active) and strain‐insensitive (electrode) parts on a single device.

**Figure 3:**
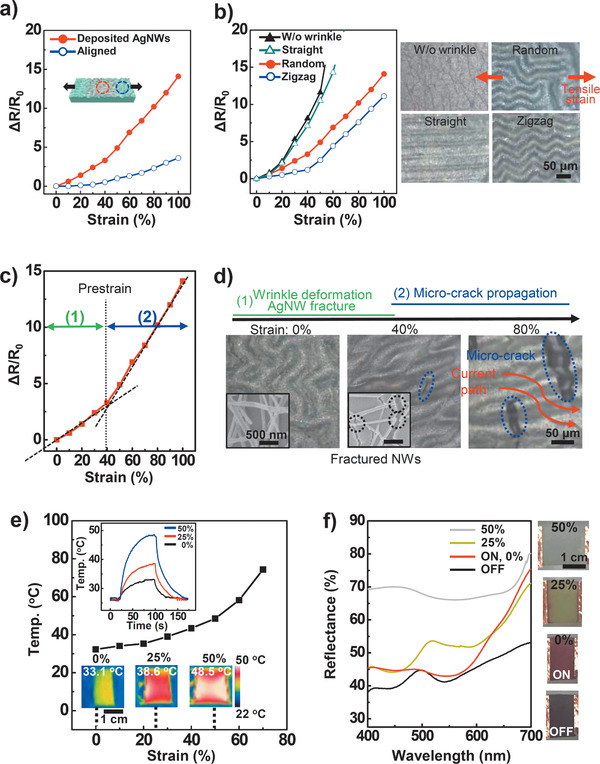
Strain‐responsive performances. a–c) Normalized resistance (Δ*R/R*_0_) changes versus uniaxial strain for prepared samples of a) aligned and deposited AgNWs on randomly wrinkled structure, b) randomly deposited AgNWs on various wrinkle geometries (random, zigzag, straight, and flat) (insets: OM images of four cases in wrinkle geometries), and c) optimized performance for stretchable strain sensor. d) Deformation of AgNWs under tensile strain up to 80% with OM and SEM images. e) Temperature changes of the user‐interactive strain sensor in freestanding state as a function of tensile strain. Insets: generated heat change images obtained with an IR camera. f) UV–vis spectroscopy data of the various colors under different strains. Insets are images of color changes recorded with an optical camera.

Figure 3b indicates that the sensitivity and stretchability of the randomly deposited AgNW strain sensors can be affected by both the geometry (e.g., random, zigzag, straight) and size of the wrinkle patterns (Figures S4–S6, Supporting Information). To obtain highly sensitive and stretchable strain sensors using the wrinkled substrate, random and small surface wrinkles are desirable. The strain‐sensitive part exhibits two stages of resistance change under tensile strain (Figure 3c). In the first stage, as the tensile strain increases to the pre‐strain (40%) used to fabricate the wrinkled structures, the surface wrinkles are deformed in a straight line along the strain direction to absorb the stress (Figure 3d). However, the nano‐sized cracks in the oxide layer on the PDMS substrates grow larger and wider,^[^ ^27^ ^]^ fracturing the AgNWs adhered to the substrate. The AgNW fractures increase the electrical resistance because of disconnection of the current paths. At strains greater than the pre‐strain level (>40%), the microcracks generated in the wrinkled substrate further reduce the current path and slightly increase the resistance.

The heating properties of the device were characterized by measuring the time‐dependent temperature as a function of tensile strain under a constant current of 0.04 A (Figure 3e). The PDMS layer covers the conductive and strain‐sensitive AgNW layer, acting as a thermal insulator against the atmospheric environment. Consequently, the surface temperature of the device can be controlled via strain from room temperature (26 °C) to specific thermotherapy temperatures (33.1 °C for 0%, 38.6 °C for 25%, and 48.5 °C for 50% strain) with a low applied voltage from 0.4 to 0.6 V (i.e., an average low power consumption of 0.02 W). The high strain sensitivity and mechanical stability (Figures S7 and S8, Supporting Information) of the stretchable device show that our fabrication method can be used to create a temperature‐tunable heater with applied mechanical strain.

To visualize the heat response to the tensile strain, we adopted a composite of three thermochromic dyes dispersed in PDMS, which enabled a reversible transition activated by different temperatures (from blue to colorless at 31 °C, from magenta to colorless at 35 °C, and from yellow to colorless at 41 °C) between two states of the lactone rings: 1) a low‐energy colored state with an open ring chain; and 2) a higher‐energy colorless state with a closed ring chain.^[^ ^6^ ^]^ When the three thermochromic dyes are mixed together, the color of the mixture is black. As the temperature of the composite film increases above 31 °C, the blue dye becomes transparent, and magenta and yellow remain. After that, when the temperature of the device rises to 35 °C under higher tensile strain, the magenta color disappears, and the device turns yellow. Finally, when the strain is higher, the temperature rises to 41 °C, the color of the device becomes white. In other words, each color disappears independently, and finally no color remains. We characterized the optical tunability of this composite film by measuring its UV–vis reflectance properties as a function of the tensile strain under the same electric current (Figure 3f). The released film without the current was almost black because of light absorption over the entire visible wavelength range as a result of the mixing of three dye colors. As the device was extended to 50%, the color changed from red (reflected wavelengths of 600–700 nm) to yellow (500–550 nm) to white, reflecting all visible wavelengths of light. These results suggested that both the heat and color of the device could be controlled via the tensile strain.

The integrated stretchable device can be attached to a finger joint or wrist for various applications such as human motion detection^[^ ^15^ ^]^ (Figure S9, Supporting Information) and thermotherapy^[^ ^18^ ^]^ (**Figure** **4**a). We designed the temperature range from released state to fully stretched one, attached to the human skin of joints. For clinical applications, the required temperature is above 40 °C, possibly between 40 and 45 °C and maintained for at least 5 min, which is considered sufficient to significantly increase tissue extensibility.^[^ ^28^ ^]^ However, as mentioned above, low‐temperature burns in human skin can be caused by critical temperature and duration time.^[^ ^23^ ^]^ Therefore, the operating temperature of the device on a finger joint skin is designed to be below about 48 °C, which prevents sudden low‐temperature burns with wearable applications. Bending the fingers increased the electrical resistance as the device was deformed, leading to heat generation under a constant and low current bias (0.04 A) from the power supply. The color changes of the optical and thermal images corroborated the heat generation by the device achieved by increasing the degree of bending (Figure 4b). To measure the effect of its thermotherapeutic rehabilitation on the joint during repeated folding (for 50 s) and unfolding (for 10 s) of the index finger,^[^ ^29^, ^30^, ^31^ ^]^ we performed a multichannel surface electromyogram (EMG) test on the forearm with and without the as‐prepared device (Figure 4c and Supporting Information). After 5 min of exercise, the EMG signal was increased by only thermotherapy during repeated folding and unfolding of the index finger (with about an 1.5 s cycle), which was attributed to the increase in the range of motion achieved by elongating the extensibility of the connective tissue because of heat. Moreover, the solution‐based fabrication method enables the device to be fabricated in a large size (6 cm × 8 cm) and with an intuitive design for application to a wrist (Figure 4d; Figure S10, Supporting Information). The “hot” indicator on the device during wrist joint flexion could help prevent low‐temperature burns as well as aid rehabilitation from many wrist diseases such as carpal tunnel and De Quervain syndrome.^[^ ^32^, ^33^, ^34^ ^]^ Wrist disease is a repetitive strain injury due to repetitive movements, sustained force, awkward postures, and other factors.^[^ ^35^ ^]^ Four pathological mechanisms have been suggested for this tendinitis disease: decrease in tendon elasticity; friction between tendon and tendon sheath; tendon fatigue; and increase in mechanically induced local temperature. For these reasons, the management of tissue elasticity or extensibility is essential to prevent or rehabilitate this kind of disease. Utilizing heat and stretch is the most effective method for increasing tissue extensibility.^[^ ^19^ ^]^ Therefore, the use of our therapeutic device on a frequently used wrist allows heat and stretching to work simultaneously by joint movement, which results in the increase of tissue extensibility as well as the avoidance of repetitive strain injury.

**Figure 4:**
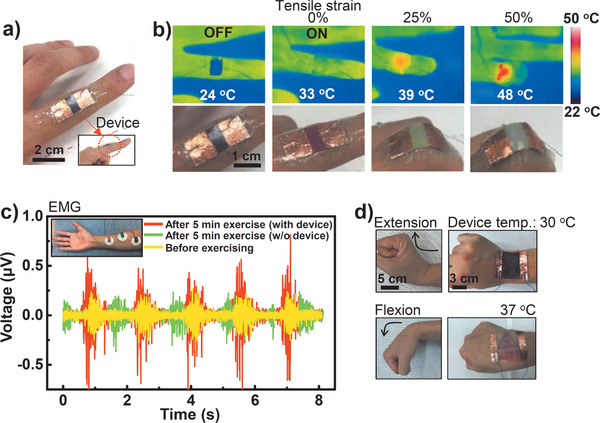
a) Photograph of the real device attached to the index finger. b) Heat and color changes of the stretchable device observed with IR and optical cameras. c) EMG signals with finger motions with and without device before and after exercise. Inset: positions of the EMG detection electrodes on the forearm. d) Large‐scale application of the device to joint movement (extension and flexion) of the wrist.

In conclusion, we demonstrated an ultrastable, stretchable, thermochromic, and thermotherapeutic device using strain‐responsive AgNW networks and thermochromic dyes in an intrinsically stretchable PDMS elastomer, which functions as both a substrate and a binder. Spontaneous patterning of AgNWs onto PDMS surfaces with surface energy‐patterned wrinkles enables control of the electrical performance of stretchable devices such as electrodes or active parts. This approach can be extended to prepare oxidation‐resistant devices by using noble metal nanowires.^[^ ^36^, ^37^ ^]^ Furthermore, a thermochromic film on a stretchable strain sensor with the same current bias can undergo a color change under different external tensile strains. The high sensitivity and stretchability of the device enable it to adaptively interface with living tissue. We speculate that the device mounted on the finger and wrist joints can be used in various applications such as a user‐interactive motion detector and a thermotherapy device. The device changes its temperature and color at different levels of joint flexion, effectively controlling the amount of heat transfer to the muscles, ligaments, and tendons of the joint as well as preventing skin burns. Integrating the stretchable strain sensor and a joule‐heater enables further application in enhancing the extensibility of injured tissue for rehabilitation patients.

## Conflict of Interest

The authors declare no conflict of interest.

## Supporting information

Supporting Information
