# Supplementary material for: User‐Interactive Thermotherapeutic Electronic Skin Based on Stretchable Thermochromic Strain Sensor
Source: Adv Sci (Weinh). 2020 Jun 8;7(17):2001184. doi: 10.1002/advs.202001184 (PMC7507701; doi:10.1002/advs.202001184)
Supplement: Supplementary file 1 — Supporting Information [file ADVS-7-2001184-s001.pdf]

Supporting Information

User-Interactive Thermotherapeutic Electronic Skin Based on Stretchable Thermochromic Strain Sensor

Giwon Lee, Geun Yeol Bae, Jong Hyun Son, Siyoung Lee, Seong Won Kim, Daegun Kim, Seung Goo Lee*, and Kilwon Cho*

Department of Chemical Engineering,

Pohang University of Science and Technology, Pohang, 37673 (Korea)

Department of Chemistry,

University of Ulsan, Ulsan, 44610 (Korea)

I. Supplementary Methods

I.1. Preparation of Patterned Silver Nanowire (AgNW) Morphology on Wrinkled PDMS Substrate

The PDMS substrate was prepared with a PDMS prepolymer and a curing agent (Sylgard 184, Dow Corning, weight ratio 20:1). The square (30 mm × 30 mm) PDMS film (~0.5 mm thick) was biaxially stretched, then exposed to UVO (Jelight 42-220, 28 mW cm^{-2}) for 50 min (**Figure S1**). The UVO-treated PDMS film was placed in a vacuum desiccator with trichloro(1H, 1H, 2H, 2H-perfluorooctyl)silane (80 µl) for 3 h with a mask of polyethylene terephthalate (PET) to selectively control the surface energy.[1–4] The pre-strain was simultaneously removed from every axial direction, yielding a random wrinkled PDMS substrate. A silver nanowire (AgNW, 0.01 g/ml in water base) (ACS materials) solution was

dropped on the wrinkled substrate. After evaporation of the droplets, the nanowires were aligned or deposited on PDMS substrates with different surface energies.

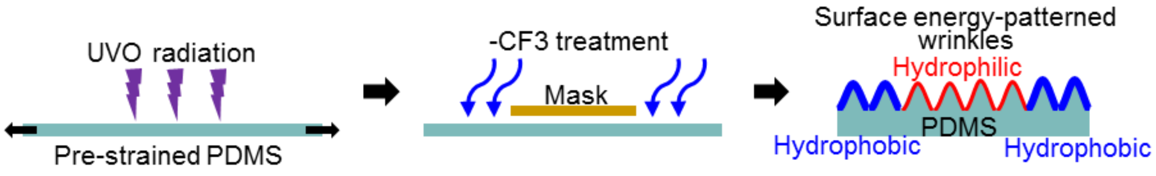

Figure S1. A schematic diagram for the hydrophilic/hydrophobic pattern control

I.2. Preparation of Various Types of PDMS Wrinkle Geometries

PDMS wrinkles with three geometries: straight, zigzag, and random were fabricated by using different pre-strain conditions. The straight pattern was fabricated by uniaxially elongating the PDMS film, exposing it to UVO, then releasing the stress. The zigzag pattern was prepared by biaxially stretching the PDMS film, exposing it to UVO, and sequentially releasing the biaxial strain. The random wrinkling pattern was fabricated by simultaneously releasing the strain.[4]

I.3. Fabrication of the User-Interactive Strain Sensor

The PDMS-based thermochromic layer was spray-coated on the randomly deposited AgNWs on the random wrinkled PDMS substrates. The thermochromic layer is composed of PDMS and three types of thermochromic dyes such as blue, magenta, and yellow. Eutectic gallium-indium (Sigma-Aldrich) and copper wires were placed on the aligned AgNW pattern to measure the change in resistance and apply a current bias.

I.4. Sample Characterization

The sample morphologies were characterized using field emission scanning electron microscopy (FESEM) (Hitachi S-4200), and optical microscopy (OM) (Axioplan Zeiss). The electrical properties of the strain sensor, current-voltage (*I*–*V*) curves, and current bias were measured using a Keithley S4200 instrument. The electrical properties of electromyography (EMG) signals were characterized with a PhysioLab p800 system. All procedures were approved by the Research Ethics Committee of Pohang University of Science and Technology in South Korea (PIRB-2020-E017). Written informed consents were obtained from all subjects.

II. Evaporation Phenomena of the AgNW Solution on the Chemically Patterned PDMS Wrinkles.

The movement of three-phase contact line (TCL) is determined by various factors such as the surface energy, solvent and solid temperature, and substrate roughness.[5–8] In this study, we modified the surface energy of the substrate. The TCL tended to move on the hydrophobic surface and remain fixed on the hydrophilic surfaces as the AgNW droplet evaporated (**Figure S2**). After evaporation was complete, the dried droplet pattern could be distinguished from the hydrophobic and hydrophilic surfaces.

A contact angle meter/CCD camera (Femtobiomed SmartDrop plus) and optical microscopy (Axioplan Zeiss) were used to observe the TCL behavior during drying (**Figure S3**). When the AgNW droplet was placed on the hydrophobic surface (water contact angle 120°), the TCL could easily move toward the center of the droplet due to the low interaction between the liquid and solid substrate. Simultaneously, the TCL shape formed overspilling droplets and elongated filaments moving at different speeds between the peaks and troughs of

the wrinkles.[2] The elongated filament lengthened as the evaporation proceeded, and the AgNWs aligned in the same direction as the filaments. However, if the droplet was deposited on the hydrophilic surface (water contact angle of 85°), high interaction with the substrate fixed the TCL during liquid evaporation. These phenomena caused the AgNWs to be randomly deposited on the PDMS wrinkles.

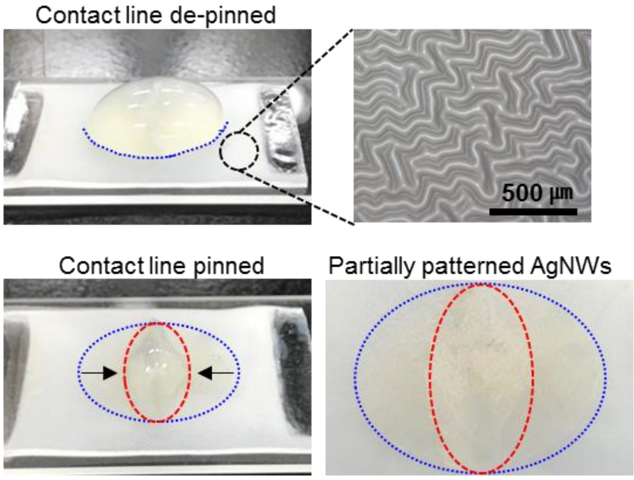

Figure S2. Photographs of the evaporation of the AgNW solution on the PDMS wrinkles

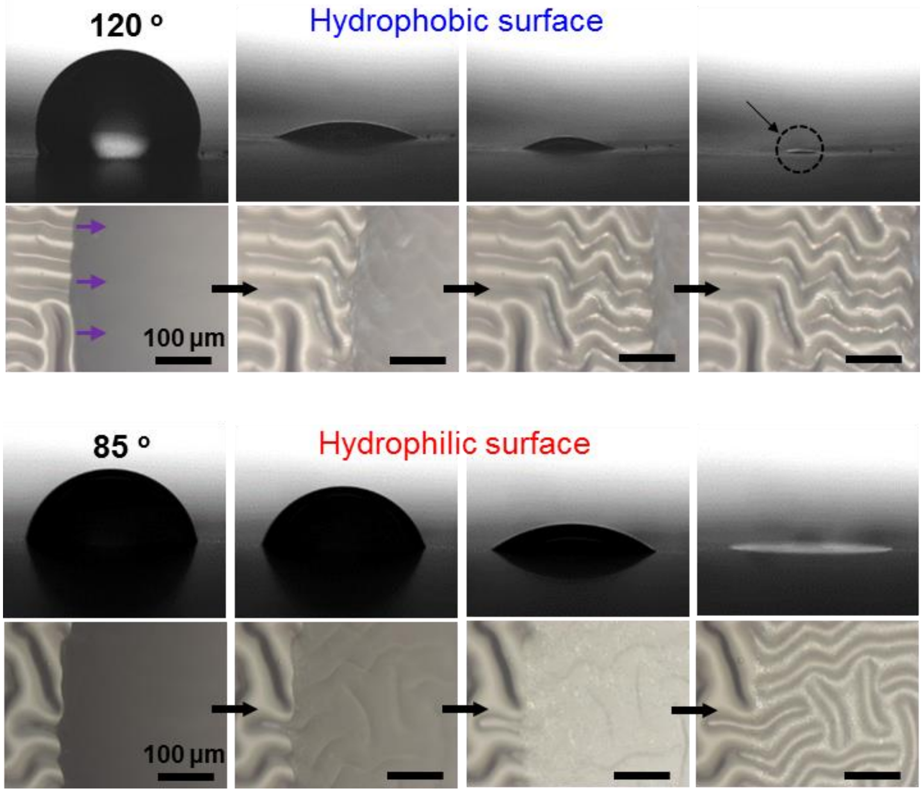

Figure S3. OM images of the contact line movement (a) and pinned state (b) during AgNW solution evaporation.

III. Mechanism of AgNW Film and PDMS Wrinkle Deformation under Tensile Strain

III.1. Geometrical Effect of Various Types of PDMS Wrinkles

To quantify the electrical properties of various AgNW deposition types on different wrinkle patterns, we also fabricated diverse wrinkle patterns such as zigzags and straight lines (**Figure S4**). These diverse substrate types indicate that the resistance for each PDMS wrinkle structure increase respectively and distinguishably. Critical points for crack initiation varied. Without the wrinkle and straight geometry on the PDMS, the electrical resistance increased steeply with the induced strain. These structures are unable to deform the surface patterns to

compensate for the mechanical stress under tensile strain, which directly induces microcracks even for small applied strains. With the random and zigzag structures, the aforementioned mechanism was followed, but the critical strain that micro-cracks start to generate is dissimilar due to the regularity of the pattern, so that means the random structure is a combination of straight and zigzag patterns. When external stress is applied to these substrates, the zigzag pattern has greater capacity to endure the stress by straightening the zigzag.[4] Although the sensitivity of the resistance change under mechanical stress is not topmost high, when applying the stretchable strain sensor, the devices should react linearly to resistance changes under tensile strain increases. Therefore, the random structure is suitable for sensor applications.

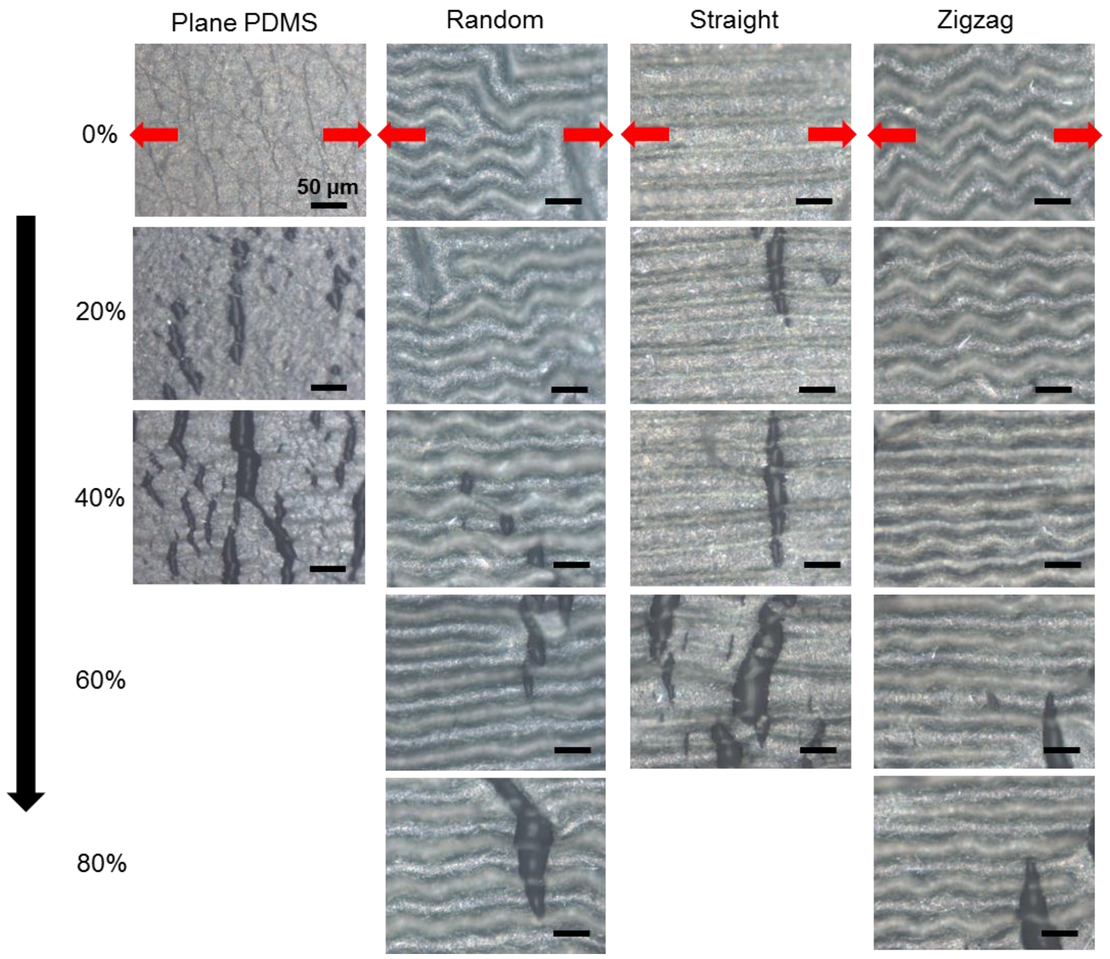

Figure S4. OM images of microcrack deformations of the wrinkle structure including plane, random, straight, and zigzag shapes under tensile strains reaching 80%.

III.2. Dimensional Effect of the Wrinkle Size

We also changed the fabrication conditions such as the UVO exposure time (50, 70, and 90 min). The wavelength and amplitude of the PDMS wrinkles increased with the UVO exposure. Increasing the amplitude of the wrinkles reduced the applied stress on the peak of the structure, leading to slight deformation of the AgNW percolation on the peak. With COMSOL simulations, the distribution of the applied tensile stress in the wrinkles is also shown with strains ranging from 0% to 100%, and fitted with the electrical data (**Figure S5**). These phenomena lower the sensitivity of the strain sensor (**Figure S6**).

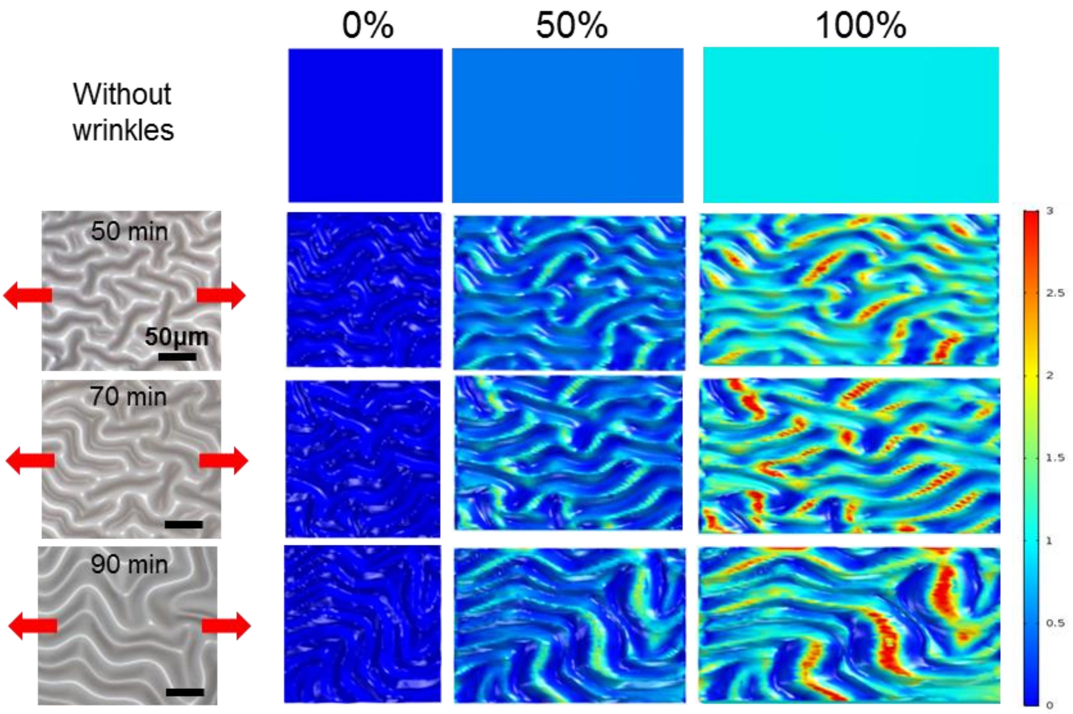

Figure S5. Optical microscopy images of the different sized wrinkles (left). COMSOL

simulation data of changes in morphology with the stress applied to the wrinkles (right).

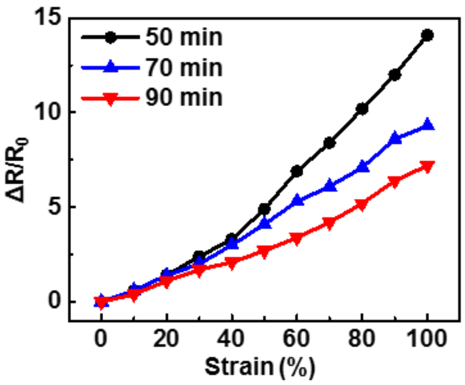

Figure S6. Change in normalized resistance ($\Delta C/C_0$) versus uniaxial strain for different sized PDMS wrinkles

III.3. Mechanical Stability of the Device

Figure S7 shows resistance changes in the random patterns of the wrinkled substrate during up to 1,000 repeated strain cycles involving stretching to 50% applied strain and releasing to 0%. Our stretchable strain sensor exhibits reversible electrical and mechanical stability after 1,000 stretching cycles, with slight fluctuation of the resistance variation. Moreover, temperature change under repeated tensile strain from 0% to 50% was measured to provide an evidence for thermal stability of this device (**Figure S8**). The trends with two cases, electrical and thermal stability, are reasonably fit under repeated external stimuli.

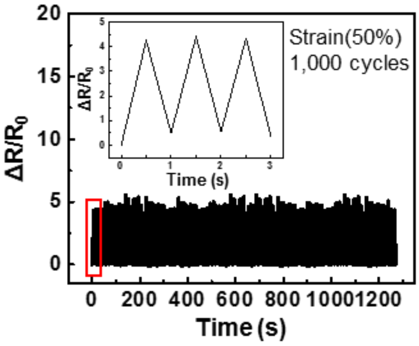

Figure S7. Normalized resistance ($\Delta R/R_0$) of the randomly wrinkled structure as a function of applied uniaxial strain over 1,000 cycles.

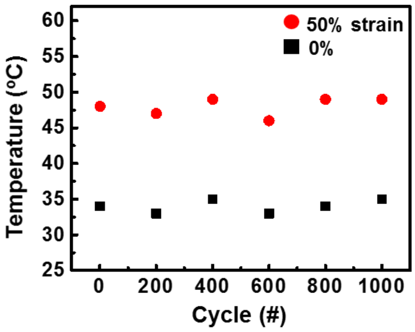

Figure S8. Temperature changes with repeated cycles of stretching-releasing.

IV. Motion Detection with the Stretchable Device

Figure S9 shows that the stretchable device attached to a human finger measured finger motion and visualized the degree of finger flexion. When the device on the finger was deformed by increased tensile strain with respect to finger flexion, the device resistance increased simultaneously (e.g., 2 corresponds to low tensile strain, 5.2 to medium, and 8.1 to high).

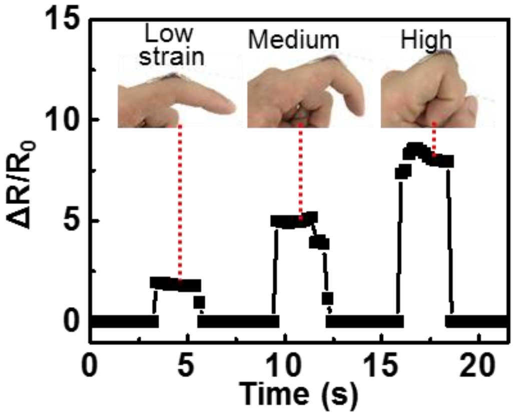

Figure S9. Detection of finger motion with resistance change.

V. Preparation for EMG Signal Detection

The electromyography (EMG) signal was detected by a digital physiograph system (PhysioLab P800). When each part of the human body deforms, the muscles near body joints are also transformed by elongating or shortening the connective tissue fibrils. The electric signal is induced by the deformation, which is EMG. The stretchable device was mounted on the index finger, and three cables for EMG detection were attached to the forearm.

VI. Large Scale Application of the Stretchable Device for Thermotherapy on a Wrist

The stretchable thermotherapeutic device was also fabricated in a large size, 6 cm× 8 cm, and applied to the edge of a wrist (**Figure S10**). The daily disease such as De Quervain syndrome frequently occur on the inner part of a wrist. Therefore, this disease can be prevented or rehabilitated by using this thermotherapeutic device in the correct position. With a wrist movement, deformation of the stretchable device induces a resistance change, followed by a variation in heat generation and color modification. This allows thermal therapy and provides heat information for users.

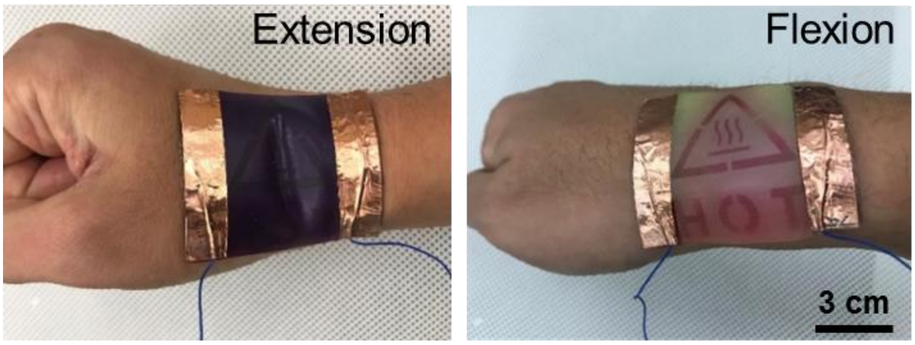

Figure S10. Optical camera images of the device deformation under wrist movement from extension to flexion.

References

[1] S. G. Lee, D. Y. Lee, H. S. Lim, D. H. Lee, S. Lee, K. Cho, *Adv. Mater*. **2010**, 22, 5013.

[2] S. G. Lee, H. Kim, H. H. Choi, H. Bong, Y. D. Park, W. H. Lee, K. Cho, *Adv. Mater*. **2013**, 25, 2162.

[3] S. G. Lee, H. S. Lim, D. Y. Lee, D. Kwak, K. Cho, *Adv. Funct. Mater*. **2013**, 23, 547.

[4] G. Lee, S. G. Lee, Y. Chung, G. Y. Bae, S. Lee, S. Ryu, K. Cho, *Adv. Electron. Mater*. **2016**, 2, 1600158.

[5] T. D. Blake, *J. Colloid Interface Sci*. **2006**, 299, 1.

[6] Y. Pomeau, *C. R. Mécanique* **2002**, 330, 207.

[7] S. Daniel, M. K. Chaudhury, J. C. Chen, *Science* **2001**, 291, 633.

[8] Z. Yoshimitsu, A. Nakajima, T. Watanabe, K. Hashimoto, *Langmuir* **2002**, 18, 5818.
